# Evaluation of Serum Selenium Status by Age and Gender: A Retrospective Observational Cohort Study in Western Romania

**DOI:** 10.3390/nu13051497

**Published:** 2021-04-28

**Authors:** Teofana Otilia Bizerea-Moga, Laura Pitulice, Otilia Bizerea-Spiridon, Tudor Voicu Moga

**Affiliations:** 1 Department XI of Pediatrics—1st Pediatric Discipline, “Victor Babeș” University of Medicine and Pharmacy Timișoara, Eftimie Murgu Sq no 2, 300041 Timișoara, Romania; bizerea.teofana@umft.ro; 2 1st Pediatric Clinic, “Louis Țurcanu” Children’s Clinical and Emergency Hospital, Iosif Nemoianu 2, 300011 Timișoara, Romania; 3 Department of Biology-Chemistry, West University of Timişoara, Pestallozi 16, 300115 Timişoara, Romania; otilia.bizerea@e-uvt.ro; 4 Laboratory of Advanced Researches in Environmental Protection, Oituz 4, 300086 Timişoara, Romania; 5 Department VII of Internal Medicine—Gastroenterology Discipline, “Victor Babeș” University of Medicine and Pharmacy Timișoara, Eftimie Murgu Sq no 2, 300041 Timișoara, Romania; moga.tudor@umft.ro; 6 Gastroenterology and Hepatology Clinic, “Pius Brînzeu” County Emergency Clinical Hospital, Liviu Rebreanu 156, 300723 Timișoara, Romania

**Keywords:** selenium, human serum, oxidative stress, age stratification, fertility, young population

## Abstract

Selenium, residing in a series of selenoproteins, plays an important role in both female and male reproductive function. Of particular significance for reproduction is the antioxidant glutathione peroxidase (GPx), a main selenoenzyme, whose level is regulated by the availability of Se in the body. We hypothesized that changes in Se status, closely related to GPx activity, would result in an increased risk of reproductive dysfunction in individuals. We retrospectively investigated the serum selenium (SeS) concentrations of 1264 apparently healthy people, aged 16–89 years, from Western Romania. The general analysis revealed a non-normal SeS distribution with a median SeS of 100.26 ± 18.32 μg/L and a significant difference in SeS levels between age groups. The analysis of the young group (16–35 years) revealed that up to 50% of individuals did not reach the SeS threshold corresponding to maximum GPx activity (80 μg/L), and a significant imbalance between the genders was apparent when looking at SeS values outside the range. Our results correlated with the general diminished reproductive ability registered in Romania during the last few years. Serum selenium content proves to offer a proper reflection of the fertility competence of the young population, and its monitoring is important for guiding dietary adjustments and attaining normal reproductive function.

## 1. Introduction

In its incredible complexity, the human body incorporates a very small percentage (0.01–0.02%) of microelements (Se, Fe, Co, Ni, Cr, Zn, V, Cu, Mn, Mo, and I), which are engaged in all body functions [1,2,3]. Among these trace elements, selenium (Se) accounts for about 13–20 mg, representing a concentration of 0.2–0.3 μg/g body weight [4].

Along with other micronutrients, such as oligo-elements, vitamins, and antioxidants, Se is a vital element for the body’s health, and its low or high intake can lead to serious disorders [5]. Selenium is strongly involved in the body’s immune function residing in a series of selenoproteins, which are important components of the antiviral, antiproliferative, antioxidant, and anti-inflammatory defense systems [5,6]. It is part of the selenoproteins that regulate a number of fundamental physiological processes, such as thyroid hormones metabolism and DNA synthesis [7]. Because selenoproteins prevent oxidative degradation of lipids and maintain redox homeostasis, Se positively intervenes in preventing diseases strongly correlated with oxidative stress, such as cardiovascular disease [8,9]. This micronutrient is also an enzymatic cofactor of some selenium proteins engaged in various functions of the central nervous system, including cognitive performance, memory, and motor coordination. For this reason, Se is thought to play a beneficial role in preventing neurodegenerative disorders, including Parkinson’s and Alzheimer’s disease, amyotrophic lateral sclerosis, and epilepsy [10].

Selenoproteins play an important role in both female and male reproductive function. In females, a series of selenoproteins, including glutathione peroxidase (GPx), thioredoxin-reductase (TR), and selenoproteins H, P, and S, mediate important processes related to postovulatory remodeling of the endometrium, growth and follicle maturation, protection of the dominant ovarian follicle, the normal progress of gametogenesis, the normal development of embryonic and extraembryonic tissues, and the normal growth of embryos. Reduction of the excess reactive oxygen species (ROS) also helps in preventing pre-eclampsia and other pregnancy-related hypertensive conditions [11]. In males, various studies indicate that reproductive and hormonal organ functions depend, to a large extent, on the status of selenium in the body. This element is necessary for testosterone biosynthesis, genesis, normal development, and the motility of sperm, as well as the protection of semen against DNA damage [12,13].

Several studies have revealed that both short- and long-term Se deficiency can have serious consequences for the genital tract, reproductive system, and fetus [14]. In women, Se deficiency may lead to an increased incidence of conditions such as acute or chronic inflammation of the uterus (metritis) or mammary glands (mastitis), ovarian cysts, infertility, placental retention, spontaneous abortion, heart and skeletal muscle damage in the fetus, and even fetal death, as well as increased susceptibility to infections of the mother and newborn [9]. In men, Se deficiency is displayed as oligospermia due to impaired spermatogenesis, decreased sperm motility and ability to fertilize, and an increased number of abnormal male sex cells as a consequence of the disruption of the integrity of their membranes due to oxidative stress [15]. On the other hand, although some authors consider that reproduction is not affected by Se excess, nonclinical studies have shown that Se overdoses or chronic exposure, even to nontoxic doses of this element, lead to biochemical disturbances of enzymes and hormones and disturb not only female fertility but also the morphology, number, and motility of sperm [16].

The prevention of Se-related health disorders and, in particular, of reproductive system function can be achieved through early and careful monitoring of the population, mainly of young people, followed by their diet adjustment and supplementation with Se as needed.

There are no national studies in Romania that have assessed the status of Se in the general population or that have investigated its association with reproductive competence. Therefore, the study aimed at determining the status of Se in Romanian individuals underlining that an unbalanced micronutrient level poses a risk to their health in general and to their reproductive capacity in particular. The study also evaluated the blood Se levels of healthy women and men from Western Romania, at the age of maximum fertility, in order to reveal if their daily diet provided an adequate intake of this trace element to ensure a suitable reproductive function.

## 2. Materials and Methods

### 2.1. Subjects

The retrospective observational study included 1264 apparently healthy subjects of 16 to 89 years of age from the western part of Romania. The study participants were selected from the subjects who presented at the blood sample collection points of the Bioclinica Medical Analysis Centre (referred to as Bioclinica), from January 2017 to December 2019, for Se testing based on the request of their physician. A subject is considered to be healthy if no diagnosis is yet established by a physician. If the individual experiences some symptoms, physicians request a detailed investigation, including analyses indicative of oxidative stress (e.g., selenium and other antioxidants). Bioclinica is the first private medical analysis clinic in Romania operating its collection and analysis centers around the country but mostly covering the western region. It also has a broad network of collaborators in Romania to which it provides services for Se analysis. Bioclinica obtained the signed consent from all subjects regarding the processing and further use of their personal data. However, the collected data of study participants were provided by Bioclinica as an anonymized database, based on a collaboration agreement with “Victor Babes” University of Medicine and Pharmacy from Timisoara. The study was approved by the Ethics Committee for Research of “Victor Babes” University of Medicine and Pharmacy from Timisoara.

The following were considered grounds for exclusion: the use of any therapy with Se dietary supplements or following a special diet, patients with confirmed pathological and chronic diseases, any type of contraceptive therapy, and pregnant women. Chronic diseases and pregnancy influence Se status by increasing oxidative stress by either pathological or physiological mechanisms [17]. High estrogen levels induced by contraception and pregnancy also modify Se metabolism by increasing selenoprotein P expression and activity [18,19,20]. The subjects were divided by age into three main groups: 16–35 years (young age group), 36–65 years (middle-aged group), and over 65 years (elderly group). The group of interest for the maximum fertility (16–35 years) was then broken down by gender.

### 2.2. Selenium Analysis

To determine selenium serum (SeS) concentrations, Bioclinica collected venous blood samples from subjects under fasting conditions and analyzed them with the ICPMS-2030 Inductively Coupled Plasma Mass Spectrometer by Shimadzu (Shimadzu Europa GmbH, Duisburg, Germany). Determinations were made according to the instructions for the use of the laboratory equipment (Laboratory Test Handbook). The ICPMS calibration with an internal standard was employed. A twelve-point calibration standard was generated using a multielement quality control standard, 100 mg/L in 5% nitric acid for 28 elements, ARISTAR^®^ (VWR International, Radnor, PA, USA), Reference 85006.186. The limit of detection (LOD), defined as 3 times the standard deviation (3 s) of the blank, was 0.0231 μg/L, and the limit of quantification (LOQ), defined as 10 s of the blank, was 0.0770 μg/L. Precision was expressed as a percentage of coefficient of variation (CV%), which was 5.6%. ClinChek^®^ Serum Control lyophilized Levels 1 and 2 (RECIPE Chemicals + Instruments GmbH, München, Germany) were used as the reference and control samples. The human-derived multielement serum control materials enabled quality assessment at two concentration levels. Prior to analysis, the control materials were reconstituted according to the manufacturer’s protocol and processed similarly as described for the serum samples.

### 2.3. Statistical Analysis

To evaluate the differences between the study groups stratified by age and gender, descriptive statistics were expressed using median values (with the range of values presented) and the median absolute deviation (MAD), as the SeS values were not normally distributed. A two-sample z-test was applied to proportions of people with SeS values within the investigated reference intervals. The data collected for the group of interest (i.e., subjects at maximum fertility age) were also statistically analyzed with the Mann–Whitney U test where appropriate. The difference between genders and SeS values stratified by the relevant intervals of concentration was also examined. A value of *p* < 0.05 was considered statistically significant. All statistical tests were two-tailed. IBM SPSS Statistics for Windows (version 27.0) was employed.

## 3. Results

### 3.1. Serum Selenium Levels of the General Population from Western Romania

Within a three-year period (1 January 2017–31 December 2019), 1264 people aged 16 to 89 years met the inclusion criteria and were included and analyzed in this retrospective study.

The analysis of the general population revealed a non-normal SeS distribution with a median SeS of 100.26 ± 18.32 µg/L. In 2017, in a total of 230 subjects, SeS values ranged from 51.09 to 219.66 μg/L, with a median of 97.59 ± 17.34 μg/L. In 2018, 419 subjects were investigated, and the range of SeS concentrations was 37.64–222.93 μg/L, with the median of 91.69 ± 18.93 μg/L. The SeS concentrations of the 615 subjects analyzed in 2019 were in the range of 23.80–299.00 μg/L, with the median of 109.46 ± 27.39 μg/L. The range and the median SeS of the studied population by age are given in Table 1.

Generally, SeS concentrations were positively associated with subjects’ age. The median values of SeS in subjects above 66 years of age were up to 31% (in 2019) higher than those of younger subjects and also more unevenly distributed.

**Figure 1:**
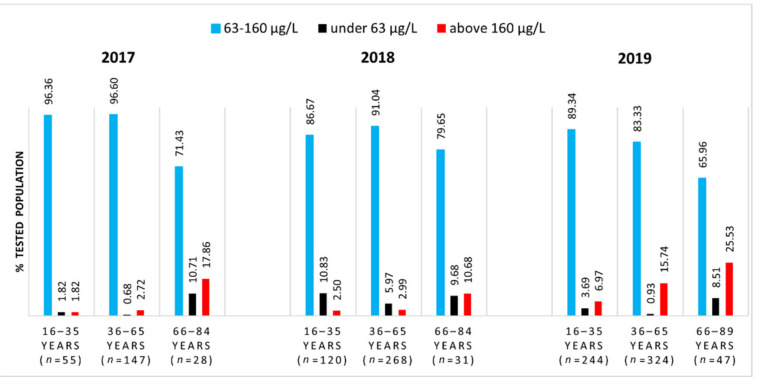
Distribution of tested population according to the SeS reference range of 63–160 μg/L.

At first, the SeS values were examined against the reference range recommended by the instructions for the use of laboratory equipment (63–160 μg/L). Figure 1 illustrates the distribution of the tested population according to the SeS reference range indicated by Bioclinica Medical Analysis Centre.

Figure 1 shows that over the years, although the number of tested people is increasing, the proportion of people with SeS values within the reference interval is slightly decreasing for all age groups. While every year, the proportions of people from young and middle-aged groups with SeS values within the reference interval do not differ significantly, the proportion of people from the elderly group is significantly lower than that for the other age ranges. For example, comparing the proportions of people from the elderly group and the middle-aged group, the differences become statistically significant: *z* = 4.655 (2017), *z* = 1.989 (2018), and *z* = 2.844 (2019). Outside the reference interval, approximately 10–25% of SeS concentrations are found above the higher limit (160 μg/L), and only up to 10% of them are below the lower limit (63 μg/L). This is to say that as the population increases in age, the SeS concentration moves toward higher values.

Furthermore, two other reference intervals were retrieved from the literature for the assessment of selenium status among the tested population: (i) a 70–130 μg/L range, which is the most often used for a healthy adult [21,22,23], and a (ii) 80–120 μg/L range, considered optimal for maximum GPx activity [24,25]. An unaltered GPx enzyme complex system ensures optimal functioning of the human organism and regulates the reproductive function. Consequently, the fertility of both men and women is beneficially influenced.

**Figure 2:**
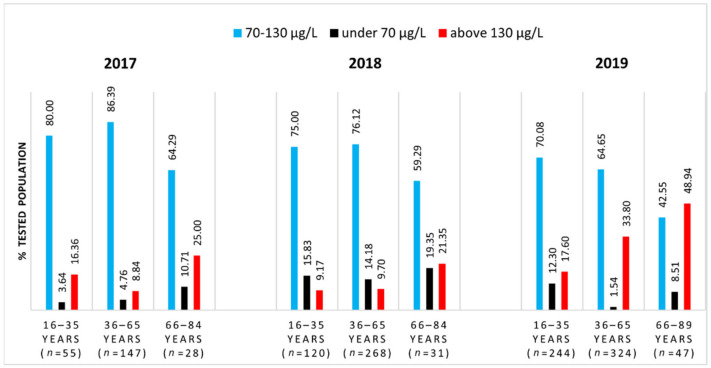
Distribution of tested population according to the SeS reference range of 70–130 μg/L.

Figure 2 indicates the percentages of the investigated population with SeS values within the reference interval of 70–130 μg/L, which is considered representative for a healthy adult.

Figure 2 illustrates the same slight decreasing trend over the years of the proportions of people with SeS values residing within this newly considered reference interval (in blue). The difference between these proportions in young and middle-aged groups is again not statistically significant, but it becomes significant when comparing either group with the elderly group (*z* = 2.844 in 2017, *z* = 2.032 in 2018, and *z* = 2.081 in 2019).

In the elderly group, people are more likely to have SeS values higher (in red) than the reference. Cumulatively, outside the reference interval, approximately 20–50% of SeS concentrations are found above the higher limit (130 μg/L), and up to 20% of them are below the lower limit (70 μg/L).

Upon comparing Figure 1 and Figure 2, it can be observed that for each age range, as long as the reference interval becomes more restrictive, the proportion of people with SeS values within that interval decreases by approximately 10–20%.

**Figure 3:**
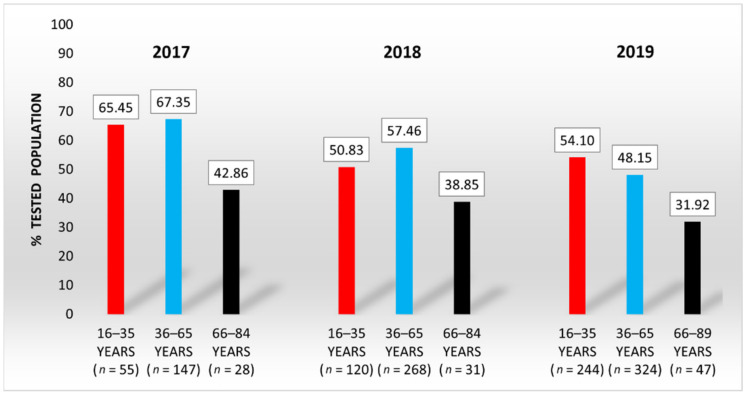
Distribution of tested population according to the SeS reference range of 80–120 μg/L.

Figure 3 indicates the percentages of the investigated population with SeS values within the interval of maximum GPx activity, 80–120 μg/L.

The data in Figure 3 demonstrate that the difference between the proportions of the middle-aged group and the elderly group is statistically significant: *z* = 2.466 (2017), *z* = 1.974 (2018), and *z* = 2.086 (2019). Data analysis confirms that the SeS values of elderly people exceeding the upper limit of the optimal range of maximum GPx activity are considerably more frequent than the values under the lower limit of this interval. As such, the proportions of tested elderly with SeS values exceeding the upper limit (120 μg/L) were 32.14% (2017), 22.58% (2018), and 55.32% (2019).

**Figure 4:**
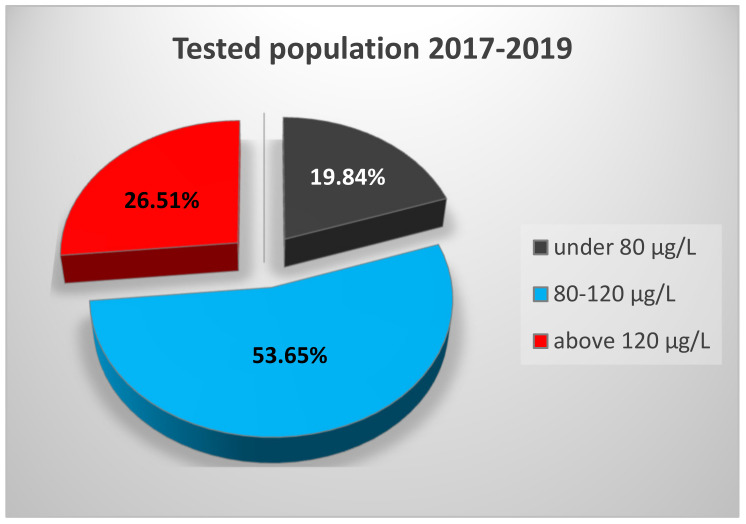
Distribution of tested population compared to the SeS reference interval of 80–120 μg/L.

Considering the overall distribution of the tested population for the entire study period (Figure 4), one can note that only 53.65% of the population has SeS concentrations within the optimal range for maximum GPx activity, regardless of age. However, one-third (33.82%) of them are young people at the age of maximum fertility.

### 3.2. Serum Selenium Levels of a Young Population from Western Romania

**Figure 5:**
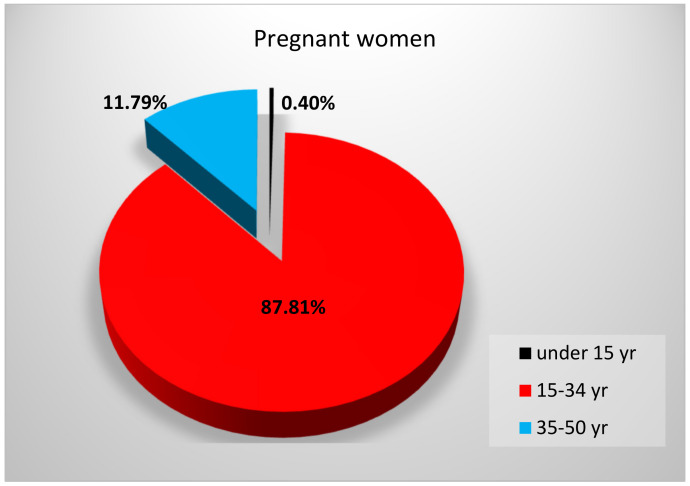
Percentages of Romanian pregnant women by different age categories in 2017.

Based on the 2017 National Report of the Population Health Status, in Romania, the majority of live births (approximately 75%) came from mothers of 20 to 34 years of age. Additionally, the share of live births from mothers under 20 years of age was high (approximately 10%) compared to the European Union level of only 2.9% [26]. The same report revealed that out of the total 128,931 pregnancies, 87.81% occurred in women of 15 to 34 years of age (Figure 5). The same tendency was observed in the following years, up to 2019 [27].

This range largely overlaps with the age range defined in our study as representing the young population. Thus, we considered that the young people aged between 16 and 35 years whom we investigated in the present study are at the age of maximum fertility. According to both the European Society of Human Reproduction and Embryology (ESHRE) and the American College of Obstetricians and Gynecologists (ACOG), fertility starts to decline when women reach their mid-30s [28,29]. Male fertility also declines between 30 and 40–45 years of age due to changes in semen quantity and quality [30,31].

For the 3-year study period, the SeS concentrations of 419 young subjects were examined and correlated with their fertility status. This group represents one-third of the study population, which corresponds to the demographic structure of the Romanian general population [32,33]. The subjects were divided based on gender into two groups as follows: 349 female subjects and 70 male subjects. Their SeS concentration range and median are presented in Table 2.

The number of tested subjects rises from one year to another, suggesting an increasing interest of both patients and physicians regarding Se assessment for the exploration of patients’ reproductive health. However, the number of tested women is roughly five times higher than the number of tested men (83.29% vs. 16.69%), with the highest gender discrepancy in 2019 (88.52% vs. 11.48%). This tendency might reside in the general perception that, in women, endocrinological and gynecological disorders are much complex and generate more fertility issues. As such, when a fertility concern emerges in a couple, the woman is most probably the first to be tested.

**Figure 6:**
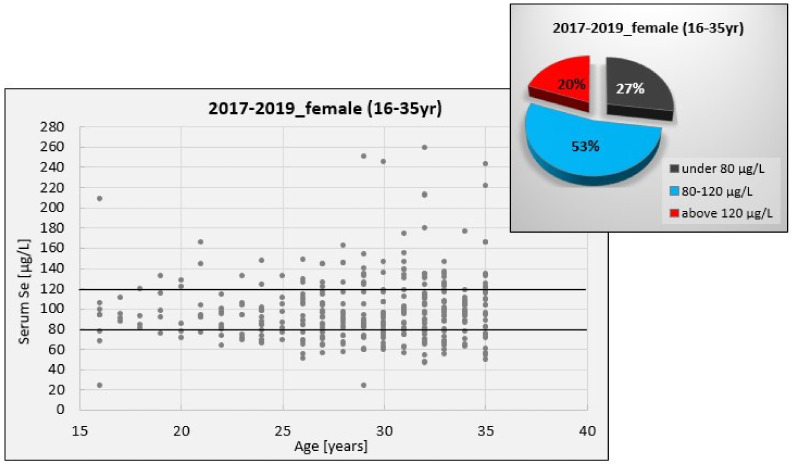
Distribution of SeS values in the young female population compared to the reference range of 80–120 μg/L.

**Figure 7:**
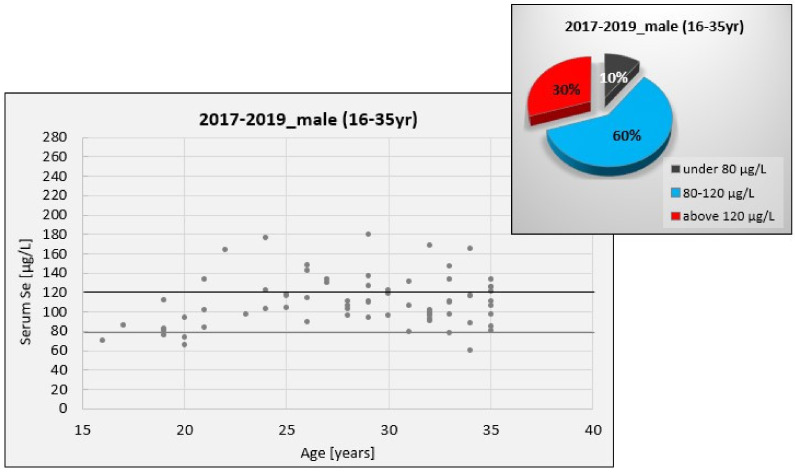
Distribution of SeS values in the young male population compared to the reference range of 80–120 μg/L.

Considering the gender analysis presented in Table 2, female subjects register a distribution of SeS values on a larger interval than male subjects. This is illustrated in Figure 6 and Figure 7, which show the distribution of SeS values in a young female and male population, respectively. These figures also show the distribution of SeS values against the reference range (80–120 μg/L), for which the level and activity of GPx are considered to be maximum.

Table 2 also reveals that the median SeS value of female subjects is lower than that of male subjects. This comes as a result of numerous female subjects with low to marginal Se status, which might be translated into a Se deficit for women when compared to the interval of maximum GPx activity (27.22% of SeS values are under the threshold, 80 μg/L). By contrast, the SeS values of male subjects in the investigated age category are less dispersed considering the interval of maximum GPx activity (MAD_male_ = 17.52 vs. MAD_female_ = 22.22).

## 4. Discussion

### 4.1. Serum Selenium Levels of the General Population from Western Romania

The assessment of Se status and intake in humans is mostly based on the investigation of its concentration or selenoproteins’ concentration/activity in the blood and related components. The Se concentration in whole blood and erythrocyte selenium reflect the long-term status, while the serum/plasma Se indicates the short-term status [25,34,35,36]. The serum/plasma Se status, plasma concentration of selenoprotein P, and plasma GPx activity are influenced by dietary intake and Se supplementation, and consequently, they may constitute nutritional selenium biomarkers for this trace element. Geographical variation in food sources and people’s lifestyles affect Se dietary intake. The differences in dietary patterns are reflected by very large variations in plasma and serum Se concentrations reported in the literature, measured according to geographical area, age, and sex [5,34,37,38].

In this retrospective observational study, we investigated the SeS concentrations in people from Western Romania in order to estimate whether their daily diets sufficiently contribute to the provision of this microelement essential for human health. We found evidence of a positive correlation between SeS concentration and population age, in agreement with other published studies [39,40,41,42]. However, other authors found a negative correlation [43,44], or no correlation, between Se concentrations and age in the general population [37,45,46]. Different age ranges and characteristic dietary profiles as well as different health conditions of the sample population may have triggered these discrepancies.

While the range of measured SeS concentrations was larger every year, the proportion of people with SeS values within the considered reference intervals decreased for all age categories, with the proportion of elderly clearly lower than for other age ranges. SeS values higher than the upper limit of all reference intervals under discussion were considerably more frequent in the elderly group than the values under the lower limit.

This tendency of high values found in the elderly might be due to their traditional diet rich in cereals and baked products, meat/meat products, eggs, milk, and dairy products, while the younger generation is oriented toward a more modern diet focused on fish, fruits, and vegetables. Nonsmoking and low alcohol consumption behavior of the elderly in Romania may also account for their elevated Se levels [47,48]. In addition, several metabolic and physiological pathways, including excretion function are known to be altered during the aging process, contributing to an accumulation of micronutrients such as Se. Yet, this “improved” Se profile has been shown to have a beneficial effect on aging and age-related manifestations [41,49,50]. Although a negative association or even no association between Se intake and metabolic syndrome has been reported [51], the risk of developing this cluster of disorders may increase in people with elevated Se levels [52,53,54,55]. Since high SeS values (>120 μg/L) were recorded in more than 25% of our studied population (Figure 4), there is a need for meticulous monitoring of this population group in line with an adequate diet and controlled administration of Se supplements to reduce potential undesirable effects.

Approximately 20% of our tested subjects (Figure 4) did not reach the Se threshold of maximum GPx activity (80 μg/L). There are several studies revealing that low levels of SeS express inflammatory reactions leading to a subsequent increase in the consumption of Se-dependent proteins, with serious consequences for the ROS–antioxidant balance. The oxidative stress, indicated by low SeS values, may be linked to a series of autoimmune inflammatory-based pathologies [56]: autoimmune thyroid disease [57,58,59], vitiligo [60,61], rheumatoid arthritis [62], and systemic lupus erythematous [63]. Thus, SeS status can be considered an appropriate biomarker for these types of pathologies. Low Se status has been also shown to be involved in the etiology of cardiovascular diseases and a variety of cancers [41,64,65]. Furthermore, the fact that up to 50% of young subjects (16–35 years) did not reach the Se level of maximum GPx activity may trigger an altered reproductive function, revealed afterward in low fertility rates.

In the case that the dietary patterns and lifestyles of these people at risk do not ensure an optimal Se level, further intervention with Se supplements and proper monitoring is required.

### 4.2. Serum Selenium Levels of a Young Population from Western Romania

In the present study, we found that SeS concentrations were within the reference range of 80–120 μg/L (corresponding to the GPx maximum activity) only for 50–60% of the young subjects. For the remainder, women had a more pronounced tendency to have SeS values below this range than men (27% vs. 10%), indicative of a serious imbalance between genders with consequences on their reproductive function. This is in accordance with the findings of low fertility rates reported in Western Romania [26]. According to the Health Status of the Romanian Population for 2017, the starting year of our study, the South West and West regions registered the lowest fertility rates (number of live births per 1000 women of childbearing age, 15–49 years), which could have further contributed to a birth rate below the national average and the greatest deficiency of natural growth in this part of the country. Changes in the birth rate can occur not only because of changes in fertility rates but also because of structural changes in the population. The report also mentions a demographic decline and an impairment of population age structure, signaling a constant decreasing trend for the population aged 15–59 years and an increasing trend for the population over 60. This, in fact, is a vicious circle, as the continuous decrease in population and its aging is primarily due to the severe birth rate decrease. Compared to European countries, the birth rate in Romania is decreasing more intensely [26]. In this context, ensuring a proper reproductive function might play an important role in Romania’s demography.

Aside from the various factors that influence the low birth and fertility rates registered in Romania, the current study sought to assess whether the level of Se in young people corresponds to a suitable reproductive function and, furthermore, whether this parameter might be a proper biomarker for people’s reproductive health. When looking at the link between selenium and female/male fertility, almost all authors agree that the status of micronutrients in the body, especially selenium, strongly influences reproductive performance [5,66,67]. Each year, young women exhibited slightly lower Se levels than young men but there were no significant statistical differences between genders. Likewise, with a few minor exceptions [45,68], other results from biomonitoring studies suggest that SeS levels are similar in men and women, regardless of geographical area [36,69,70,71,72]. Considering the interval of maximum GPx activity, Se concentration in human serum can be subdivided into three categories: low level (below 80 µg/L), normal level (between 80 and 120 µg/L), and high level (above 120 µg/L). According to this classification, our results suggest that the current Se status of the young female population from Western Romania is somewhat normal to low, while that of the male population is normal to high. This difference is better observed when comparing the gender groups outside the range. We found a significant difference between proportions of females and males with SeS values below the range (*z* = 3.064). Their lifestyle/dietary behavior might be one of the reasons [73,74]. Women from Western Romania (a high socioeconomic region), especially the young ones, tend to consume food with low nutritional quality, as they pay special attention to their fitness and beauty, without considering the low micronutrient content of this diet. It has been revealed that daily intakes of Se < 55 µg (the recommended dietary allowance for a healthy individual) were associated with an increased risk of sporadic anovulation [75]. Another reason might reside in the Se–estrogen relationship. Nonclinical studies investigating the mechanism of action suggested that estrogen status affects Se metabolism by modulating a protein encoded by the Sepp1 gene that regulates Se transport [20]. In healthy women, Se concentration and GPx activity have been found to have a linear relationship with estrogen during the menstrual cycle [76], a case in which a woman’s fertility holds a normal status. In women with a Se deficit, reproductive competence can be compromised, significantly decreasing their chances of conception. Furthermore, even in the event of a successful pregnancy, Se deficiency increases the risk of pregnancy-induced hypertensive disorders, preterm birth, and miscarriage [77]. While still in the womb of Se-deficient mothers, the fetus is exposed to intrauterine growth restriction and small weight for gestational age [78,79,80,81]. The newborn is also affected by insufficient Se levels, which, combined with iodine deficiency, have been linked to endemic cretinism [82]. As such, Se deficiency triggers a chain reaction that follows women in all reproductive stages. Although increasing levels of estrogen throughout pregnancy modulate selenoprotein P and subsequently affect tissue distribution of Se, inadequate Se intake will ultimately lead to low plasma selenoprotein P levels [18,19,20]. The downregulation of selenoprotein P encoded by Sepp1 will promote oxidative stress throughout pregnancy [83,84,85].

On the other hand, studies in both animals and humans suggest that Se is also important for the biosynthesis of testosterone in men, although the mechanism is not yet clear. It has been revealed that Se positively influences testosterone production and spermatogenesis as well as the increase in sperm motility and morphology [86,87,88,89,90]. An in vitro study [91] revealed that Se could modulate the proliferation and apoptosis of Leydig cells, which are known to produce testosterone. As such, an optimal level of Se proved to enhance testosterone production, whereas a higher Se level triggered a reduced proliferation and increased apoptosis of Leydig cells. In our young male study group, 30% of men had higher SeS values than normal, which could have caused impairments of their reproductive function.

Overall, both Se-deficit and Se-surplus may result in reproductive dysfunction of young individuals, and our results correlate with the general diminished reproductive ability registered in Romania during the last years. We agree that SeS is indicative of reproductive competence and is a useful tool for identifying the grounds of fertility decline in otherwise healthy individuals (without pathological diseases). Our study’s strengths include the relatively high number of subjects and the longitudinal analysis of SeS, which provides real-world data at the regional level needed to understand the current situation and to better tailor more informative subsequent studies. The study’s limitations include its observational nature and the unavailability of information regarding the fertility of individuals, which could have conferred greater usefulness for public health. We also relied on participant self-reporting of their health status (i.e., unknown healthy issues), diet, and mineral supplementation when applying the exclusion criteria, which may have generated a more heterogenous population sample and influenced the results.

## 5. Conclusions

The reproductive competence of otherwise healthy women and men (without pathological diseases) may clearly be affected by a variety of factors (e.g., age, reproductive system disorders, lifestyle, socioeconomic status, and environmental factors). Regardless of the targeted pathway, the fluctuations occurring at this level are linked to Se status in the body. Serum Se content proves to offer a proper reflection of the fertility status of the young population, and its monitoring is of great importance for guiding dietary adjustments and attaining normal reproductive function. To this end, the awareness and education of populations currently undergoing economic development and fertility decline can be integrated into the management and minimization of associated risks.

## Figures and Tables

**Table 1:** Range and median of SeS concentrations in all subjects from Western Romania by age.

Year	Age Range (yr)	Number of Subjects	Median SeS ^1^ (μg/L)	SeS Range (μg/L)
2017	16–35	55	96.11 ± 15.00	51.09–165.59
	36–65	147	97.27 ± 16.08	58.54–219.66
	≥66	28	98.66 ± 25.77	52.32–184.60
2018	16–35	120	89.00 ± 17.49	46.63–176.46
	36–65	268	92.35 ± 18.70	42.69–222.93
	≥66	31	99.56 ± 24.85	37.64–205.37
2019	16–35	244	98.45 ± 24.20	23.80–259.00
	36–65	324	115.00 ± 27.18	51.25–299.00
	≥66	47	129.00 ± 33.50	48.33–281.00

^1^ Values of serum selenium (SeS) are presented as median ± median absolute deviation (MAD).

**Table 2:** Range and median SeS concentration of young subjects (16–35 years) from Western Romania by gender.

Gender	Number of Subjects			Total Number of Subjects	Median SeS ^1^ (μg/L)	SeS Range (μg/L)
	2017	2018	2019			
Female	42	91	216	349	94.15 ± 22.22	23.80–259.00
Male	13	29	28	70	106.05 ± 17.52	60.05–180.00

^1^ Values of SeS are presented as median ± MAD.

## Data Availability

The data are not publicly available due to reasons of privacy.

## References

[1] Aliasgharpour M., Farzami M. (2013). Trace Elements in Human Nutrition: A Review. Inter. J. Med. Invest..

[2] Prashanth L., Kattapagari K., Chitturi R., Baddam V., Prasad L. (2015). A Review on Role of Essential Trace Elements in Health and Disease. J. Dr. Ntr. Univ. Health Sci..

[3] Wada O. (2004). What are Trace Elements? Their Deficiency and Excess States. Jpn. Med. Assoc. J..

[4] Al-Fartusie F., Mohssan S. (2017). Essential Trace Elements and Their Vital Roles in Human Body. Indian J. Adv. Chem. Sci..

[5] Rayman M.P. (2012). Selenium and Human Health. Lancet.

[6] Croft L., Lu J., Holmgren A., Khanna K. (2007). From Selenium to Selenoproteins: Synthesis, Identity, and Their Role in Human Health. Antioxid. Redox Signal..

[7] Mehdi Y., Hornick J.L., Istasse L., Dufrasne I. (2013). Selenium in the Environment, Metabolism and Involvement in Body Functions. Molecules.

[8] Skalnaya M., Skalny A. (2018). Essential Trace Elements in Human Health: A Physician’s View.

[9] Hosnedlova B., Kepinska M., Skalickova S., Fernandez C., Ruttkay-Nedecky B., Malevu T.D., Sochor J., Baron M., Melcova M., Zidkova J. (2017). A Summary of New Findings on the Biological Effects of Selenium in Selected Animal Species-A Critical Review. Int. J. Mol. Sci..

[10] Solovyev N.D. (2015). Importance of Selenium and Selenoprotein for Brain Function: From Antioxidant Protection to Neuronal Signalling. J. Inorg. Biochem..

[11] Qazi I.H., Angel C., Yang H., Pan B., Zoidis E., Zeng C.J., Han H., Zhou G.B. (2018). Selenium, Selenoproteins, and Female Reproduction: A Review. Molecules.

[12] Alahmar A.T. (2019). Role of Oxidative Stress in Male Infertility: An Updated Review. J. Hum. Reprod. Sci..

[13] Qazi I.H., Angel C., Yang H., Zoidis E., Pan B., Wu Z., Ming Z., Zeng C.J., Meng Q., Han H. (2019). Role of Selenium and Selenoproteins in Male Reproductive Function: A Review of Past and Present Evidences. Antioxidants.

[14] Pal A. (2015). Role of Copper and Selenium in Reproductive Biology: A Brief Update. Biochem Pharm..

[15] Bleau G., Lemarbre J., Faucher G., Roberts K.D., Chapdelaine A. (1984). Semen Selenium and Human Fertility. Fertil. Steril..

[16] Chowdhury A.R., Dhundasi S. (2009). Recent Advances in Heavy Metals Induced Effect on Male Reproductive FunctioŽ A Retrospective. J. Med. Sci..

[17] Aouache R., Biquard L., Vaiman D., Miralles F. (2018). Oxidative Stress in Preeclampsia and Placental Diseases. Int. J. Mol. Sci..

[18] Hill K.E., Wu S., Motley A.K., Stevenson T.D., Winfrey V.P., Capecchi M.R., Atkins J.F., Burk R.F. (2012). Production of Selenoprotein P (Sepp1) by Hepatocytes is Central to Selenium Homeostasis. J. Biol. Chem..

[19] Xia Y., Hill K.E., Li P., Xu J., Zhou D., Motley A.K., Wang L., Byrne D.W., Burk R.F. (2010). Optimization of Selenoprotein P and other Plasma Selenium Biomarkers for the Assessment of the Selenium Nutritional Requirement: A Placebo-Controlled, Double-Blind Study of Selenomethionine Supplementation in Selenium-Deficient Chinese subjects. Am. J. Clin. Nutr..

[20] Zhou X., Smith A.M., Failla M.L., Hill K.E., Yu Z. (2012). Estrogen Status Alters Tissue Distribution and Metabolism of Selenium in Female Rats. J. Nutr. Biochem..

[21] Sheehan T.M.T., Halls D.J. (1999). Measurement of Selenium in Clinical Specimens. Ann. Clin. Biochem..

[22] Safaralizadeh R., Kardar G.A., Pourpak Z., Moin M., Zare A., Teimourian S. (2005). Serum Concentration of Selenium in Healthy Individuals Living in Tehran. Nutr. J..

[23] Fuggle S. (2018). Clinical Biochemistry Reference Ranges Handbook.

[24] Arnaud J., Bertrais S., Roussel A.M., Arnault N., Ruffieux D., Favier A., Berthelin S., Estaquio C., Galan P., Czernichow S. (2006). Serum Selenium Determinants in French Adults: The SU.VI.M.AX study. Br. J. Nutr..

[25] Millán Adame E., Florea D., Sáez Pérez L., Molina López J., López-González B., Pérez de la Cruz A., Planells del Pozo E. (2012). Deficient Selenium Status of a Healthy Adult Spanish Population. Nutr. Hosp..

[26] Cucu M.A., Cristea C., Calomfirescu C., Matei E., Ursu C., Rădulescu S., Georgescu D. (2018). Raportul Naţional al Stării de Sănătate a Populaţiei României din 2017. https://insp.gov.ro/sites/cnepss/wp-content/uploads/2014/11/SSPR-2017-1.pdf.

[27] Cucu M.A., Cristea C., Matei E., Galan A., Ursu C., Dima C., Georgescu D. (2020). Raportul Naţional al Stării de Sănătate a Populaţiei României din 2019. https://insp.gov.ro/sites/cnepss/wp-content/uploads/2020/12/Raport-Starea-de-Sanatate-2019.pdf.

[28] American College of Obstetricians and Gynecologists Committee on Gynecologic Practice and Practice Committee (2014). Female Age-Related Fertility Decline. Fertil. Steril..

[29] Baird D.T., Collins J., Egozcue J., Evers L.H., Gianaroli L., Leridon H., Sunde A., Templeton A., Van Steirteghem A., Cohen J. (2005). Fertility and Ageing. Hum. Reprod. Update.

[30] Harris I.D., Fronczak C., Roth L., Meacham R.B. (2011). Fertility and the Aging Male. Rev. Urol..

[31] Hassan M.A.M., Killick S.R. (2003). Effect of Male Age on Fertility: Evidence for the Decline in Male Fertility with Increasing Age. Fertil. Steril..

[32] National Institute of Statistics-Romania Tendinte sociale. 2019, 17. https://insse.ro/cms/sites/default/files/field/publicatii/tendinte_sociale.pdf.

[33] National Institute of Statistics-Romania (2020). Populaţia După Domiciliu* la 1 Ianuarie 2020 a Ajuns la 22 175 mii Persoane. https://insse.ro/cms/sites/default/files/com_presa/com_pdf/popdom1ian2020r.pdf.

[34] Thomson C.D., Caballero B. (2003). SELENIUM|Physiology. Encyclopedia of Food Sciences and Nutrition.

[35] Dumont E., Vanhaecke F., Cornelis R. (2006). Selenium Speciation from Food Source to Metabolites: A Critical Review. Anal. Bioanal. Chem..

[36] Noisel N., Carrier G., Bouchard M. (2014). Study of Selenium Intake and Disposition in Various Matrices Based on Mathematical Algorithms Derived from Pooled Biomonitoring Data. Int. J. Hyg. Environ. Health.

[37] Burri J., Haldimann M., Dudler V. (2008). Selenium Status of the Swiss Population: Assessment and Change Over a Decade. J. Trace Elem. Med. Biol..

[38] Rocourt C.R., Cheng W.H. (2013). Selenium Supranutrition: Are the Potential Benefits of Chemoprevention Outweighed by the Promotion of Diabetes and Insulin Resistance?. Nutrients.

[39] Rasmussen L.B., Hollenbach B., Laurberg P., Carlé A., Hög A., Jørgensen T., Vejbjerg P., Ovesen L., Schomburg L. (2009). Serum Selenium and Selenoprotein P Status in Adult Danes—8-year followup. J. Trace Elem. Med. Biol..

[40] Sánchez C., López-Jurado M., Aranda P., Llopis J. (2010). Plasma Levels of Copper, Manganese and Selenium in an Adult Population in Southern Spain: Influence of Age, Obesity and Lifestyle Factors. Sci. Total Environ..

[41] Cai Z., Zhang J., Li H. (2019). Selenium, Aging and Aging-Related Diseases. Aging Clin. Exp. Res..

[42] González-Estecha M., Palazón-Bru I., Bodas-Pinedo A., Trasobares E., Palazón-Bru A., Fuentes M., Cuadrado-Cenzual M.Á., Calvo-Manuel E. (2017). Relationship Between Serum Selenium, Sociodemographic Variables, other Trace Elements and Lipid Profile in an Adult Spanish Population. J. Trace Elem. Med. Biol. Organ Soc. Miner. Trace Elem. (Gms).

[43] Alehagen U., Johansson P., Björnstedt M., Rosén A., Post C., Aaseth J. (2016). Relatively High Mortality Risk in Elderly Swedish Subjects with Low Selenium Status. Eur. J. Clin. Nutr..

[44] Robberecht H., De Bruyne T., Davioud-Charvet E., Mackrill J., Hermans N. (2019). Selenium Status in Elderly People: Longevity and Age-Related Diseases. Curr. Pharm. Des..

[45] Kim Y.J., Galindev O., Sei J.H., Bae S.M., Im H., Wen L., Seo Y.R., Ahn W.S. (2009). Serum Selenium Level in Healthy Koreans. Biol. Trace Elem. Res..

[46] Bocca B., Madeddu R., Asara Y., Tolu P., Marchal J.A., Forte G. (2011). Assessment of Reference Ranges for Blood Cu, Mn, Se and Zn in a Selected Italian Population. J. Trace Elem. Med. Biol..

[47] Thomson C.D. (2004). Assessment of Requirements for Selenium and adequacy of Selenium Status: A Review. Eur. J. Clin. Nutr..

[48] Chen C.J., Lai J.S., Wu C.C., Lin T.S. (2006). Serum Selenium in Adult Taiwanese. Sci. Total. Environ..

[49] Alis R., Santos-Lozano A., Sanchis-Gomar F., Pareja-Galeano H., Fiuza-Luces C., Garatachea N., Lucia A., Emanuele E. (2016). Trace Elements Levels in Centenarian ’Dodgers’. J. Trace Elem. Med. Biol..

[50] Gerardo B., Cabral Pinto M., Nogueira J., Pinto P., Almeida A., Pinto E., Marinho-Reis P., Diniz L., Moreira P.I., Simões M.R. (2020). Associations between Trace Elements and Cognitive Decline: An Exploratory 5-Year Follow-Up Study of an Elderly Cohort. Int. J. Environ. Res. Public Health.

[51] Retondario A., Fernandes R., Rockenbach G., Alves M.A., Bricarello L.P., Trindade E., Vasconcelos F.A.G. (2019). Selenium Intake and Metabolic Syndrome: A Systematic Review. Clin. Nutr..

[52] Laclaustra M., Navas-Acien A., Stranges S., Ordovas J.M., Guallar E. (2009). Serum Selenium Concentrations and Diabetes in U.S. adults: National Health and Nutrition Examination Survey (NHANES) 2003–2004. Environ. Health Perspect..

[53] Lu C.W., Chang H.H., Yang K.C., Kuo C.S., Lee L.T., Huang K.C. (2016). High Serum Selenium Levels are Associated with Increased Risk for Diabetes Mellitus Independent of Central Obesity and Insulin Resistance. Bmj Open Diabetes Res. Amp. Care.

[54] Fontenelle L., Feitosa M., Morais J., Severo J., Freitas T., Beserra J., Henriques G., Marreiro D. (2018). The Role of Selenium in Insulin Resistance. Braz. J. Pharm. Sci..

[55] Lu C.W., Chang H.H., Yang K.C., Chiang C.H., Yao C.A., Huang K.C. (2019). Gender Differences with Dose-Response Relationship between Serum Selenium Levels and Metabolic Syndrome-A Case-Control Study. Nutrients.

[56] Sahebari M., Rezaieyazdi Z., Khodashahi M. (2019). Selenium and Autoimmune Diseases: A Review Article. Curr. Rheumatol. Rev..

[57] Federige M.A.F., Romaldini J.H., Miklos A.B.P.P., Koike M.K., Takei K., Portes E.d.S. (2017). Serum Selenium and Selenoprotein-P Levels in Autoimmune Thyroid Diseases Patients in a Select Center: A Transversal Study. Arch. Endocrinol. Metab..

[58] Alijani E., Abbasi N. (2020). The Effect of Selenium on Hashimoto’s Thyroiditis; Systemic Review and Metaanalysis. J. Clin. Toxicol..

[59] Bednarczuk T., Schomburg L. (2020). Challenges and Perspectives of Selenium Supplementation in Graves’ Disease and Orbitopathy. Horm. (Athens).

[60] Colucci R., Dragoni F., Moretti S. (2015). Oxidative Stress and Immune System in Vitiligo and Thyroid Diseases. Oxid. Med. Cell Longev.

[61] Fan K.-C., Yang T.-H., Huang Y.-C. (2018). Vitiligo and Thyroid Disease: A Systematic Review and Meta-Analysis. Eur. J. Dermatol..

[62] Moustafa S., Al-Tawil N., Abid F. (2015). Association of Boron, Copper, Germanium, Magnesium, Selenium and Zinc with Incidence of Rheumatoid Arthritis. Am. J. Intern. Med..

[63] Sahebari M., Abrishami-Moghaddam M., Moezzi A., Ghayour-Mobarhan M., Mirfeizi Z., Esmaily H., Ferns G. (2014). Association Between Serum Trace Element Concentrations and the Disease Activity of Systemic Lupus Erythematosus. Lupus.

[64] Bleys J., Navas-Acien A., Guallar E. (2008). Serum Selenium Levels and All-Cause, Cancer, and Cardiovascular Mortality Among US Adults. Arch. Intern. Med..

[65] Narod S.A., Huzarski T., Jakubowska A., Gronwald J., Cybulski C., Oszurek O., Dębniak T., Jaworska-Bieniek K., Lener M., Białkowska K. (2019). Serum Selenium Level and Cancer Risk: A Nested Case-Control Study. Hered. Cancer Clin. Pr..

[66] Stoffaneller R., Morse N.L. (2015). A Review of Dietary Selenium Intake and Selenium Status in Europe and the Middle East. Nutrients.

[67] Grieger J.A., Grzeskowiak L.E., Wilson R.L., Bianco-Miotto T., Leemaqz S.Y., Jankovic-Karasoulos T., Perkins A.V., Norman R.J., Dekker G.A., Roberts C.T. (2019). Maternal Selenium, Copper and Zinc Concentrations in Early Pregnancy, and the Association with Fertility. Nutrients.

[68] Wąsowicz W., Zachara B.A. (1987). Selenium Concentrations in the Blood and Urine of a Healthy Polish Sub-Population. J. Clin. Chem. Clin. Biochem..

[69] Shortt C.T., Duthie G.G., Robertson J.D., Morrice P.C., Nicol F., Arthur J.R. (1997). Selenium Status of a Group of Scottish Adults. Eur. J. Clin. Nutr..

[70] Navarro-Alarcon M., Cabrera-Vique C. (2008). Selenium in Food and the Human Body: A Review. Sci. Total. Environ..

[71] Letsiou S., Nomikos T., Panagiotakos D., Pergantis S., Fragopoulou E., Antonopoulou S., Pitsavos C., Stefanadis C. (2010). Dietary Habits of Greek Adults and Serum Total Selenium Concentration: The ATTICA study. Eur. J. Nutr..

[72] Belhadj M., Kazi Tani L.S., Dennouni Medjati N., Harek Y., Dali Sahi M., Sun Q., Heller R., Behar A., Charlet L., Schomburg L. (2020). Se Status Prediction by Food Intake as Compared to Circulating Biomarkers in a West Algerian Population. Nutrients.

[73] Pagalea A., Uta D.S.V. (2012). Romanian Consumer Lifestyle and Attitude towards Bio Products Purchase. Procedia—Soc. Behav. Sci..

[74] Voinea L., Vranceanu D., Filip A., Popescu D., Negrea M., Dina R. (2019). Research on Food Behavior in Romania from the Perspective of Supporting Healthy Eating Habits. Sustainability.

[75] Kim K., Wactawski-Wende J., Michels K.A., Schliep K.C., Plowden T.C., Chaljub E.N., Mumford S.L. (2018). Dietary Minerals, Reproductive Hormone Levels and Sporadic Anovulation: Associations in Healthy Women with Regular Menstrual Cycles. Br. J. Nutr..

[76] Ha E.J., Smith A.M. (2003). Plasma Selenium and Plasma and Erythrocyte Glutathione Peroxidase Activity Increase with Estrogen During the Menstrual Cycle. J. Am. Coll Nutr..

[77] Lucky H.S., Sajal G., Yesul K., Ashok A. (2010). Female Infertility and Antioxidants. Curr. Women’s Health Rev..

[78] Bizerea T.O., Dezsi S.G., Marginean O., Stroescu R., Rogobete A., Bizerea-Spiridon O., Ilie C. (2018). The Link Between Selenium, Oxidative Stress and Pregnancy Induced Hypertensive Disorders. Clin. Lab..

[79] Mamon M.A.C., Menodiado C.M.M., Siasu G.L., Ramos G.B. (2016). Selenium Supplementation within the Periconception Period: Influence on Maternal Liver and Renal Histoarchitecture. Asian Pac. J. Reprod..

[80] Zadrozna M., Gawlik M., Nowak B., Marcinek A., Mrowiec H., Walas S., Wietecha-Posłuszny R., Zagrodzki P. (2009). Antioxidants Activities and Concentration of Selenium, Zinc and Copper in Preterm and IUGR Human Placentas. J. Trace Elem. Med. Biol..

[81] Ba Z. (2016). Selenium in Pregnant Women: Mini Review. J. Nutr. Food Sci..

[82] McArdle H.J., Ashworth C.J. (1999). Micronutrients in Fetal Growth and Development. Br. Med. Bull..

[83] Akbaba G., Akbaba E., Sahin C., Kara M. (2018). The Relationship Between Gestational Diabetes Mellitus and Selenoprotein-P Plasma 1 (SEPP1) Gene Polymorphisms. Gynecol. Endocrinol..

[84] Hofstee P., Cuffe J.S.M., Perkins A.V. (2020). Analysis of Selenoprotein Expression in Response to Dietary Selenium Deficiency During Pregnancy Indicates Tissue Specific Differential Expression in Mothers and Sex Specific Changes in the Fetus and Offspring. Int. J. Mol. Sci..

[85] Wu H., Jia X., Zhao H., Huang Y., Liu C., Huang Z., Li S., Wang J. (2017). Identification of SEPP1 Polymorphisms is not a Genetic Risk Factor for Preeclampsia in Chinese Han Women: A Clinical Trial and Experimental Study. Medicine.

[86] Scott R., MacPherson A., Yates R.W., Hussain B., Dixon J. (1998). The Effect of Oral Selenium Supplementation on Human Sperm Motility. Br. J. Urol..

[87] Oguntibeju O., Esterhuyse J.S., Truter E. (2009). Selenium: Its Potential Role in Male Infertility. Pak. J. Med. Sci..

[88] Oluboyo A.O., Adijeh R.U., Onyenekwe C.C., Oluboyo B.O., Mbaeri T.C., Odiegwu C.N., Chukwuma G.O., Onwuasoanya U.F. (2012). Relationship Between Serum Levels of Testosterone, Zinc and Selenium in Infertile Males Attending Fertility Clinic in Nnewi, South East Nigeria. Afr. J. Med. Med. Sci..

[89] Villaverde A.I.S.B., Fioratti E.G., Ramos R.S., Neves R.C.F., Ferreira J.C.P., Cardoso G.S., Padilha P.M., Lopes M.D. (2014). Blood and Seminal Plasma Concentrations of Selenium, Zinc and Testosterone and their Relationship to Sperm Quality and Testicular Biometry in Domestic Cats. Anim. Reprod. Sci..

[90] Rezaeian Z., Yazdekhasti H., Nasri S., Rajabi Z., Fallahi P., Amidi F. (2016). Effect of Selenium on Human Sperm Parameters after Freezing and Thawing Procedures. Asian Pac. J. Reprod..

[91] Shi L., Song R., Yao X., Ren Y. (2017). Effects of Selenium on the Proliferation, Apoptosis and Testosterone Production of Sheep Leydig Cells in Vitro. Theriogenology.

